# ERAT combined with electrohydraulic lithotripsy for the treatment of chronic appendicitis with giant fecal stones in the appendix: A case report

**DOI:** 10.1097/MD.0000000000048473

**Published:** 2026-04-24

**Authors:** Chun Gao, Xiang-Xiang Zhu, Wei Wang

**Affiliations:** a Department of Gastroenterology, The 940 Hospital of Joint Logistic Support Force of PLA, Lanzhou, Gansu, China.

**Keywords:** chronic appendicitis, electrohydraulic lithotripsy, ERAT, giant appendicolith, minimally invasive treatment

## Abstract

**Rationale:**

Endoscopic retrograde appendicitis therapy (ERAT) has emerged as a minimally invasive treatment option for acute uncomplicated appendicitis. However, its application in chronic appendicitis with giant appendicoliths is rarely reported. Such cases present a significant challenge for ERAT due to the difficult removal of large, impacted fecal stones using conventional techniques.

**Patient concerns:**

A 40-year-old male presented with persistent right lower quadrant pain, tenderness, and elevated inflammatory markers.

**Diagnoses:**

Abdominal computed tomography confirmed appendiceal dilation and a giant fecal stone, leading to a diagnosis of chronic appendicitis with a giant appendicolith.

**Interventions:**

The patient underwent ERAT, during which electrohydraulic lithotripsy (EHL) was employed to fragment the large stone that was not amenable to direct extraction.

**Outcomes:**

ERAT combined with EHL successfully fragmented and removed the appendicolith, relieving the appendiceal obstruction. The patient’s abdominal pain resolved promptly postoperatively, inflammatory markers normalized, and he was discharged on the second postoperative day. During a 12-month follow-up period, the patient remained asymptomatic, with follow-up imaging showing no signs of recurrence.

**Lessons:**

ERAT combined with EHL is a safe and effective approach for managing chronic appendicitis with giant appendicoliths. This combined strategy overcomes the technical challenge of removing large, impacted stones via a minimally invasive, organ-preserving approach, avoiding the risks associated with surgical intervention. It represents a promising therapeutic alternative for these challenging cases.

## 1. Introduction

Appendiceal fecaliths are formed by mineral deposits and hardened feces, which often lead to appendiceal obstruction, which can cause ischemia, inflammation, and even gangrene, perforation, and extensive peritonitis to occur in the appendix. It has been shown that when appendicitis is caused by appendiceal fecal stone obstruction, the risk of complications is significantly higher than that of those without appendiceal fecal stone if they receive antibiotic treatment alone.^[1]^ In addition, when appendicitis is caused by an obstructed appendiceal fecalith, the risk of complications is significantly higher when treated with antibiotics alone than when there is no appendiceal fecalith.^[2]^

The appendix has long been recognized as a degenerate organ that has lost its function during evolution. However, in recent years, a large body of literature suggests that the appendix may be a reservoir or “safe house” that maintains the balance of the intestinal microbial community.^[3]^ Surgical removal of the appendix is the preferred treatment for patients with acute appendicitis or acute exacerbation of chronic appendicitis, both nationally and internationally. However, removal of the appendix is not a permanent solution, and studies have shown that appendectomy increases the risk of inflammatory bowel disease, colorectal cancer, and systemic lupus erythematosus in women.^[4-6]^

With the in-depth study of endoscopic retrograde cholangiopancreatography (ERCP), Said et al saved a patient with acute appendicitis by using ERCP in combination with electronic enteroscopy in 1995.^[7]^ Inspired by the principle of ERCP for the treatment of acute obstructive septic cholangitis, Prof Liu Bingrong performed the first endoscopic retrograde appendicitis treatment in 2009.^[8]^ There is an international consensus that the goal of acute appendicitis is to minimize the rate of negative appendectomy without increasing the incidence of complications such as perforation due to delayed diagnosis,^[9]^ and ERAT can achieve this goal by providing rapid relief of pain and preserving the appendix. However, recurrence of appendicitis is still an unresolved issue. In this paper, we report a case of chronic appendicitis combined with a huge fecal stone in the appendix was treated with endoscopic ERAT combined with liquid electrode lithotripsy, and good results were achieved. It is reported as follows:

## 2. Case presentation

**Figure 1.**
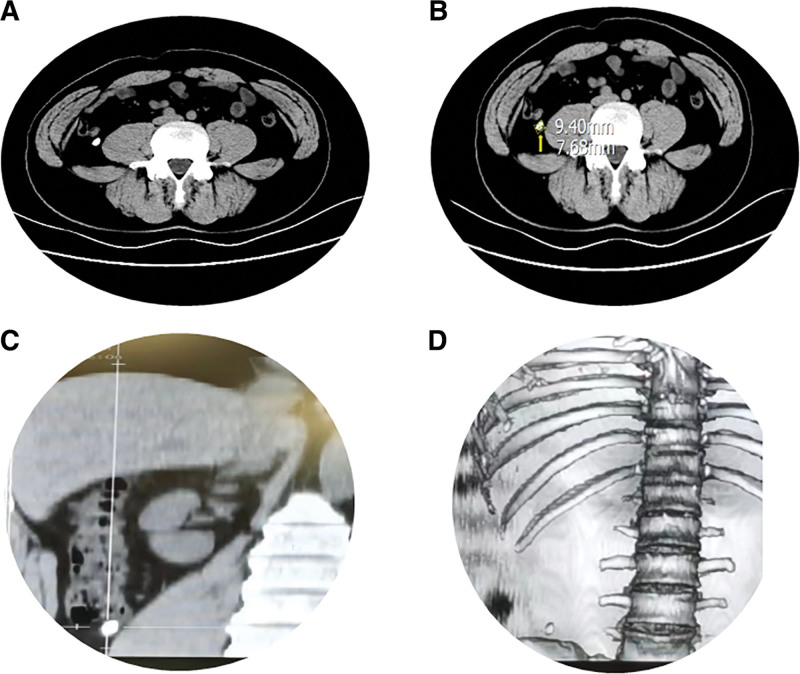
Abdominal CT scan before treatment. (A) Axial non‐contrast CT image shows a nodular hyperdense lesion in the appendiceal region of the right lower quadrant, consistent with appendicolith. (B) Axial non‐contrast CT image with linear measurements of the appendicolith. (C) Coronal reconstructed CT image clearly depicts the distal course of the appendix and the location of the appendicolith. (D) Three-dimensional volume‐rendered CT image shows the density comparison between the appendicolith and adjacent bone, suggesting the calcified characteristic of the stone. CT = computed tomography.

A 40-year-old male was admitted to the hospital with “intermittent right lower abdominal pain for 10 years, recurring for 1 day.” The patient had recurrent right lower abdominal pain 10 years ago, which was aggravated by alcohol consumption and strenuous exercise, and was discharged from the hospital after several visits to abdominal ultrasound and abdominal computed tomography (CT) suggesting chronic appendicitis and appendiceal fecaliths, and anti-inflammatory treatments because of refusal to undergo appendectomy. This time, he was hospitalized for abdominal pain that appeared again 1 day ago. CT of the whole abdomen suggested: appendiceal fecal stone, size about 8 mm × 9 mm, oval shape (Fig. 1).

**Figure 2.**
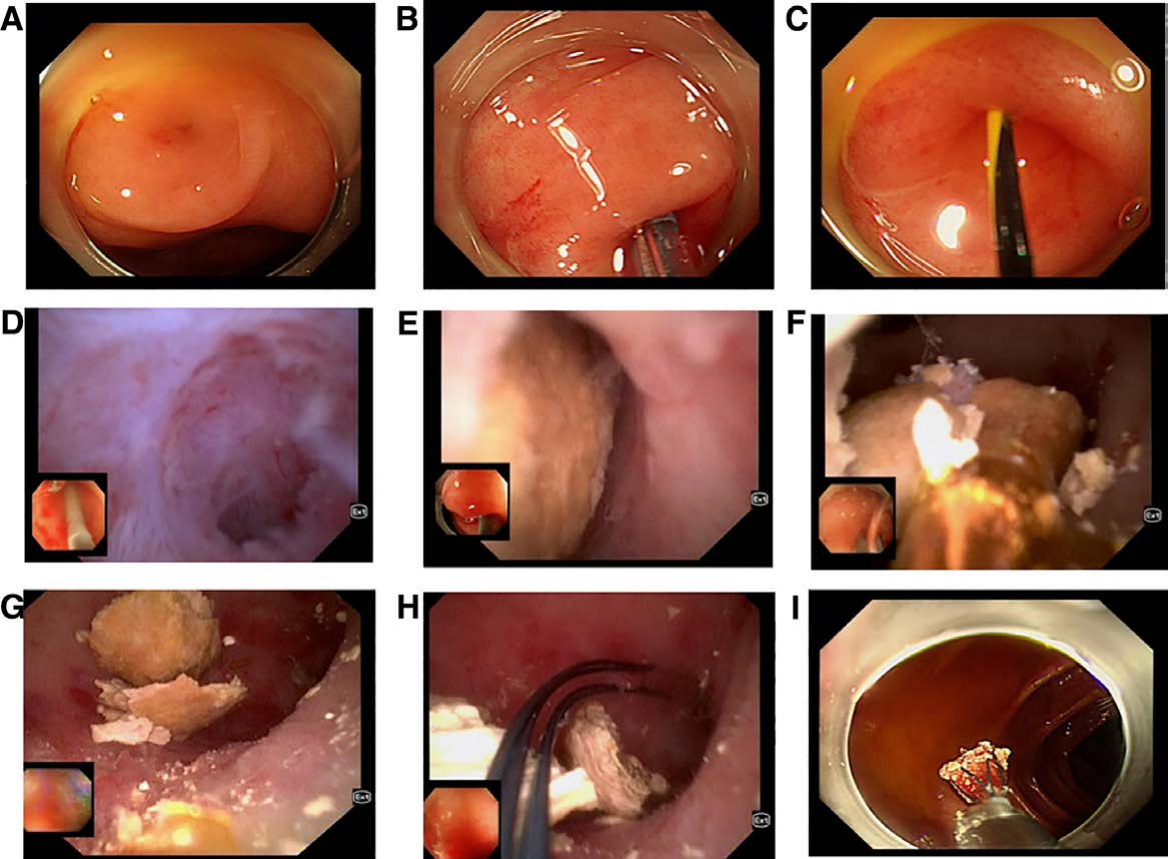
Endoscopic view and treatment process. (A) Narrow opening of the appendix, (B) guide the guide wire into the appendix and dilate the opening of the appendix with a surgical incision, (C) endoscope enters the appendiceal cavity, (D) the appendiceal cavity is tortuous and angled, (E) large stones in the appendix cavity, (F) liquid electric gravel and fragments, (G, H) appendiceal irrigation and basket stone retrieval, (I) remove the stones and end the treatment.

After full communication with the patient, ERAT was proposed. The bowel was prepared using sodium phosphate salts, and after successful intravenous anesthesia, the colonoscope (Olympus 290, Olympus Medical Systems Corporation, Tokyo, Japan) with a transparent cap was successfully entered into the cecum, and the narrowing of the appendiceal opening was observed to be nearly occluded, and the appendiceal lumen was entered using a contrast catheter with a yellow zebra guidewire, and the guidewire guided the contrast catheter into the appendiceal lumen, and after the dilation of the appendiceal opening, the NJ Minimally Invasive Biliopancreatic Imaging catheter (9 Fr) was used to enter the appendiceal lumen, and mucosal congestion in the appendiceal lumen was observed, The mucosa of the appendiceal lumen was observed to be congested, edematous and erosive, and the appendiceal lumen was tortuous, with 1 place close to an acute angle. After adjusting the direction of the biliopancreatic imaging catheter and colonoscope to pass through the place, it was observed that there was a huge yellow fecal stone in the appendiceal lumen, which was hard in texture, and could be movable in the lumen (Fig. 2).

**Figure 3.**
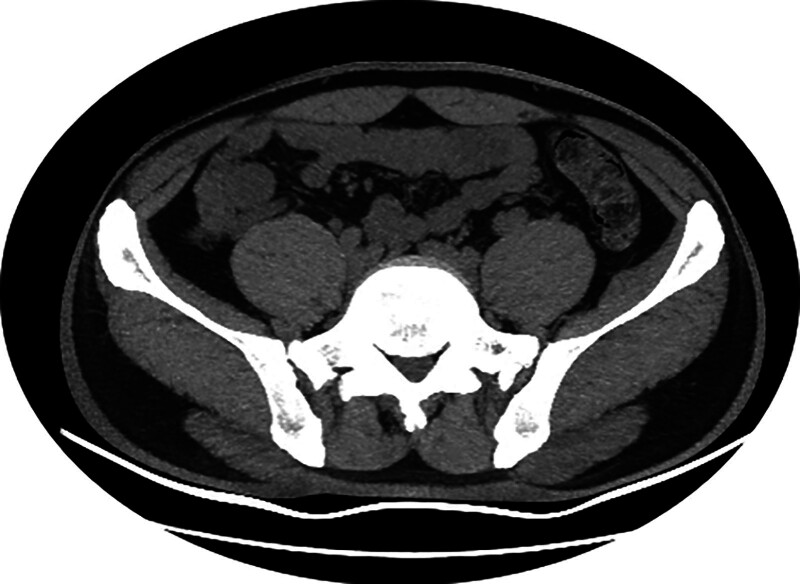
Abdominal CT scan after treatment. CT = computed tomography.

**Figure 4.**
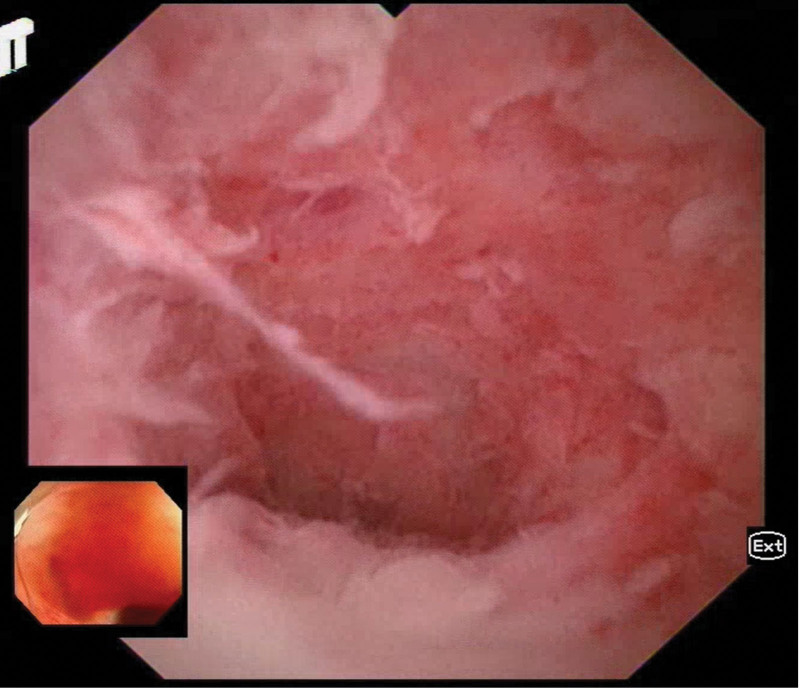
Endoscopic view after treatment.

Using liquid electrode lithotripsy (IMES in vivo microelectrode lithotripter, OOD pulse mode, energy setting 0.2 Joules, Xi’an Yuanhong Science and Technology) and duodenoscopic electrode (NDJ2210, Xi’an Yuanhong Science and Technology) to directly contact with the stone and inject water to fill the appendiceal cavity was successful in lithotripsy, and the stone was crushed into a number of small stones by several times of repetition. After repeated cleaning with saline rinsing and a special mesh basket (Poco, spyglass retrieval basket), all the stones were successfully removed, and no obvious bleeding was observed in the appendiceal cavity (Fig. 3, Supplementary Video 1). After the operation, the patient was fasted with water and kept in bed for 24 hours, and was treated with ceftriaxone for anti-infection and rehydration. The patient recovered well, resumed eating and going down to the ground 24 hours after the operation without any obvious discomfort. Abdominal CT was repeated 72 hours after the operation to observe mild enlargement of the appendix without residual stones (Fig. 4), the patient was successfully discharged from the hospital and continued to take oral cefdinir for 4 days. After a 1-year follow-up, there was no recurrence of abdominal pain (Table 1).

**Table 1:** Clinical timeline of the case.

Timeline	Key events	Examinations/interventions	Outcomes
Day 0 (admission)	Admission for recurrent RLQ pain	History taking, physical examination, abdominal CT scan	Confirmed diagnosis of chronic appendicitis with an appendicolith (8 mm × 9 mm)
Day 1	Pre-procedural preparation	Patient counseling and consent for ERAT	Decision to proceed with ERAT as a minimally invasive treatment option
Day 2	ERAT Procedure	ERAT combined with electrohydraulic lithotripsy	The procedure has been determined
Postoperative day 1 (day 3)	Inhospital recovery	Fasting, bed rest, intravenous fluids, IV ceftriaxone	Patient remained stable with no immediate post-procedural complications
Postoperative day 2 (day 4)	Continued recovery	Diet resumed, ambulation initiated	Patient tolerated oral intake well and was mobile without discomfort
Postoperative day 3 (day 5)	Predischarge assessment	Follow-up abdominal CT scan	CT revealed mild appendiceal swelling but no residual stones (Fig. 4). Patient discharged
At discharge	Outpatient management	Oral cefdinir was prescribed for 4 ds	To ensure complete resolution of inflammation and prevent infection
1-yr postoperative	Long-term follow-up	Clinical follow-up (symptom assessment)	No recurrence of abdominal pain, confirming the long-term success of the procedure

CT = computed tomography, ERAT = endoscopic retrograde appendicitis therapy, RLQ = right lower quadrant.

## 3. Discussion

Chronic appendicitis is relatively common in clinical practice, and the most typical clinical manifestations are intolerable pain in the right lower abdomen, or pain around the umbilicus, middle and upper abdomen, or may be accompanied by nausea and vomiting, and other clinical manifestations, and the body’s immunity is weakened. In addition, chronic appendicitis may also be triggered by gastrointestinal disease stimulation of appendicitis foci, with abdominal pain, abnormal rise in body temperature as the main clinical manifestations, which can make their normal life and work seriously affected.^[10]^ Chronic appendicitis can be divided into primary chronic appendicitis and secondary chronic appendicitis, the condition of intermittent recurrent episodes, the course of the disease is usually longer, prolonged, if not treated in a timely manner, it is easy to lead to acute episodes, the formation of appendiceal perforation foci, or caused by periappendiceal abscesses and peritonitis and other complications, threatening the patient’s life safety. Therefore, chronic appendicitis needs to be treated in a timely manner in order to strengthen the control of the disease, improve its clinical outcome, optimize its quality of life, promote the recovery of patients as soon as possible, and then improve the prognosis.^[11,12]^

Obstruction of the appendiceal lumen complicated by bacterial infection is the basic etiology of appendicitis, and it has been reported in the literature that about 35% of appendicitis is caused by fecal retention or fecal stone obstruction.^[13]^ The appendiceal lumen is elongated and distal to the appendix is blind. When a fecal stone becomes embedded in the appendiceal lumen, the distal end of the embedded fecal stone becomes a dead space, forming a closed-collar obstruction. As the appendix continuously secretes mucus and accumulates in the lumen, the pressure in the lumen increases, the blood flow in the appendiceal wall is impaired, the venous return is impeded and venous thrombosis occurs, and the appendix is obviously congested and edematous. Secondary bacterial infection on the basis of appendiceal obstruction makes the appendiceal lesion more serious.^[14,15]^

In recent years, with the rapid development of minimally invasive medical technology, the treatment concept of preserving the appendix has gradually gained importance. Traditional appendicitis treatments rely on open surgery or laparoscopic appendectomy, which are effective in treating appendicitis in most cases, but inevitably remove the appendix, thus losing its potential immune function and other physiological functions. ERAT, on the other hand, as a new minimally invasive treatment, offers a new treatment option for appendicitis patients. It can treat the etiology of chronic appendicitis by intubating, imaging, flushing, and placing a stent to drain the appendix, removing appendiceal fecaliths, and relieving obstruction of the appendiceal lumen, which leads to a rapid regression of appendicitis, and thus achieves the goal of to appendicitis, and thus is highly efficacious.^[16]^ As found in a meta-analysis, ERAT reduced operative time and intraoperative bleeding compared to appendectomy.^[17]^ Liu et al^[18]^ found that the advantages of ERAT over appendectomy included no skin wound, organ preservation, reduced postoperative pain, early feeding, faster recovery, fewer postoperative complications, and shorter postoperative hospitalization.

However, when chronic appendicitis is combined with a large fecal stone in the appendix, the treatment options are often challenging. Due to the large size and hard texture of the fecal stone, it is difficult to remove it completely by traditional methods, and it may lead to appendiceal perforation or peripheral abscess formation due to improper operation, which in turn increases the surgical risk. ERAT technology combined with electrohydraulic lithotripsy (EHL) provides a solution to this problem. Electrolytic lithotripsy was firstly proposed and successfully applied by foreign experts. It adopts the principle of electrolytic shock wave to carry out targeted micro-bursting of lithotripsy to break the stone into small pieces or powder, which is easy to be flushed out and discharged subsequently, and it has the advantages of shorter lithotripsy time and higher output power, etc.^[19]^ This technique is widely used in surgical operations and ERCP lithotripsy. The liquid electrode lithotripsy can partially fracture the stone, and after removing the stone fragments, the remaining large pieces can be fractured again. This is repeated so that the stone is completely removed in a single endoscopic procedure. This ensures that the image is clear and the fragments do not interfere with the field of view during lithotripsy, which improves the success rate of lithotripsy, and also reduces the number of times the stone is removed, avoiding the higher rate of postoperative complications and hospitalization costs associated with split endoscopic lithotripsy.

The combined application of ERAT and liquid electrolithotripsy in the treatment of chronic appendicitis combined with a large fecal stone in the appendix can fully utilize the synergistic effect of both. First, ERAT provides precise navigation and stable operation platform for liquid electro lithotripsy, which makes the lithotripsy process safer and more effective. Second, ERAT solves the problems that ERAT may encounter when dealing with large fecaliths and ensures the thoroughness of the treatment. The 2 complement each other and together they achieve effective treatment of chronic appendicitis combined with appendicular giant fecal stone. In the present study, ERAT was found to be successful for appendiceal lumen intubation and stone retrieval in combination with liquid electrocautery lithotripsy, and was finally found to be free of serious complications such as intraoperative and postoperative perforation. After the appendiceal fecal stone obstruction is relieved, the patient’s abdominal pain can be relieved quickly.

Although this case successfully applied the combination of ERAT combine EHL to treat chronic appendicitis with a giant appendicolith, it is crucial to recognize that this technique poses certain challenges and carries potential risks of complications and failure. First, the technical procedure itself can lead to complications. During appendiceal orifice cannulation and guidewire passage, there is a risk of appendiceal perforation, particularly when the orifice is edematous and stenotic or when a concealed perforation exists. The EHL used in this case, if performed with improper energy settings or misaligned fiber targeting the calculus, could also cause injury to the appendiceal wall. Furthermore, excessively high irrigation pressure intraoperatively may facilitate the spread of infection, potentially triggering bacteremia or intra-abdominal infection.^[20]^ Second, the possibility of treatment failure cannot be overlooked. The primary reasons for failure include: Factors related to the appendicolith: Excessively large, hard, or impacted fecaliths may not be effectively fragmented by EHL. Even after fragmentation, larger remnants might fail to be expelled, leading to incomplete treatment. Anatomical factors: severe stenosis, tortuosity, or atresia of the appendiceal lumen can prevent the endoscope from advancing, rendering ERAT unfeasible. Inadequate infection control: if the pus is too viscous or drainage is insufficient, the patient’s symptoms may persist or recur postoperatively, ultimately necessitating surgical intervention.

This study has several limitations. First, this is a single case report, which can only demonstrate the successful application of this technique in 1 specific patient and cannot provide conclusive evidence regarding its general efficacy. Second, as the use of ERAT for treating appendicitis with giant fecaliths is itself an emerging exploration, there is currently a lack of large-scale clinical studies or randomized controlled trials for comparison. Its long-term efficacy, recurrence rate, and complication rate still require validation through more prospective studies. Finally, the successful outcome in this case may be influenced by case selection bias, and the results may not be generalizable to all types of complicated appendicitis.

## 4. Conclusion

With the advancement of medical technology and the deepening of the concept of minimally invasive treatment, ERAT combined with EHL is expected to play an even more important role in the treatment of appendicitis and bring benefits to more patients. At the same time, we also need to explore and optimize the operation procedure and indication range of this technique to further improve its therapeutic effect and safety.

## Author contributions

**Project administration:** Chun Gao.

**Software:** Chun Gao, Xiang-Xiang Zhu.

**Supervision:** Xiang-Xiang Zhu, Wei Wang.

**Validation:** Wei Wang.

**Writing – original draft:** Chun Gao.

**Writing – review & editing:** Chun Gao.
